# Environmental Exposure Science and Human Health

**DOI:** 10.3390/ijerph20105764

**Published:** 2023-05-09

**Authors:** Anna M. Lavezzi, Bruno Ramos-Molina

**Affiliations:** 1 “Lino Rossi” Research Center for the Study and Prevention of Unexpected Perinatal Death and SIDS, Department of Biomedical, Surgical and Dental Sciences, University of Milan, 20122 Milan, Italy; 2 Obesity and Metabolism Laboratory, Biomedical Research Institute of Murcia (IMIB), 30120 Murcia, Spain

## 1. Introduction

Human health and environmental exposure form an inseparable binomial. Humans’ relationships with the environment are indeed a fundamental determinant of their state of health. According to the World Health Organization (WHO), among the environmental factors that pose the greatest risk to human health are: air pollution, heavy metals (such as arsenic, cadmium, lead and mercury), dioxins, pesticides and benzene [1]. Due to their degradation resistance, these and many other toxic pollutants may remain in our environments for a long time, both in confined (“indoor”) and open (“outdoor”) spaces, and can easily enter the body. The permanent absorption of these pollutants through inhalation, ingestion and/or direct skin contact can cause bioaccumulation in many tissues [2,3,4,5,6], leading to serious damage, especially related to cardiovascular and respiratory pathologies, as well as cancer, obesity (and related metabolic disorders such as diabetes) and neurodegenerative illness [7,8,9]. The WHO estimates that approximately 13 million deaths annually are attributable to environmental exposure, of which over 7 million are related to air pollution alone, particularly that associated with fine particulate matter (PM2.5, PM10) [1]. Numerous factors can be taken into consideration regarding damage to human health caused by environmental pollution, particularly during the most vulnerable stages of life, such as early childhood and pregnancy. Some of these factors are mentioned in the following sections.

## 2. Adverse Outcomes of Environmental Pollutants in Young Children

Adverse pollution-related impacts are more frequent in children in the first months of life than in adults. According to the WHO, more than 33% of cases in children under 5 occur due to environmental causes [1]. The greater susceptibility to pollutants in early childhood is most likely due to the fact that young children’s physiological detoxification mechanisms for the removal of xenobiotics from the body are not yet fully developed, giving rise to various diseases, including metabolic disorders such as obesity. In particular, a growing body of evidence has shown that some chemical toxins, known as “obesogens”, are capable of interfering with lipid homeostasis and adipocyte physiology, promoting obesity in children, a pathology that has reached epidemic levels in developed countries [10].

Furthermore, the high incidence of tumors at this age cannot be attributed, as is often the case for adults, to poor lifestyle choices (alcohol assumption, smoking, etc.), as these are incompatible with early childhood; rather, it is often attributed to the trans-placental passage of toxic substances in the prenatal period or through the mother’s milk in the first months of life [11,12,13,14]. 

It is important to underline that today, the fetal developmental phase is considered the most crucial stage in the development of health conditions not only in childhood, but also in adulthood, which is why we often speak of the “fetal origin of adult diseases” [15]. In particular, it has been shown that exposure to toxic substances during early childhood may affect the lungs, resulting in the potential development of asthma, pneumonia or chronic pulmonary disease [16,17]. These findings are of particular concern, and are well-aligned with WHO’s reports stating that more than 90% of children are breathing toxic air daily [18]. 

### 2.1. Xenobiotic Chemical Damage to the Maternal–Fetal Unit

Although the protective ability of the placenta toward the fetus is well known, numerous studies have shown that several toxic substances with which a pregnant woman comes into contact are able to easily cross this barrier and enter the bloodstream of the fetus, with consequent repercussions for its development and for the state of the child’s health after birth [19,20]. Among the xenobiotics to which fetuses may be exposed as a consequence of transplacental passage, the most common are pharmaceuticals, nicotine and cotinine (markers of tobacco smoke), and chemical substances such as pesticides [19,20,21,22,23]. These can induce greater toxicity in the fetus than in the mother due to the poor development of the prenatal detoxification system. This could lead to fetal growth delay, congenital heart defects and adverse birth outcomes, such as preterm delivery, low birth weight, or even unexpected perinatal death [24,25,26,27].

### 2.2. Pollutants in Breast Milk

Maternal exposure to air pollution during pregnancy and after delivery can lead to increased concentrations of pollutants (such as dioxins, furans, polychlorinated biphenyls, DDT, heavy metals and organochlorine pesticides) in breast milk [28]. Additionally, when pollutants are absorbed through the mother’s milk, consequences can occur not only in the short term, but also in later years; this increases the risk of the onset of chronic pathologies, mainly in the infant.

### 2.3. Maternal Smoking in Pre- and Postnatal Life

Tobacco smoke contains over 5300 compounds, including more than 70 substances that the International Agency for Research on Cancer (IARC) has classified as type 1 carcinogens (i.e., carcinogenic to humans) [29]. Despite the potentially serious harmful effects of these substances, many women continue to smoke both during pregnancy and after delivery. This habit deserves particular attention, since as frequently documented in the literature, it can be associated not only with adverse fetal outcomes (such as growth retardation, preterm birth, low birth weight and stillbirth), but also with pathologies related to the infant’s respiratory system, obesity, attention deficit disorder and autism.

Carbon monoxide (CO), one of the agents that is primarily responsible for the adverse effects of cigarette smoke if a mother smokes during pregnancy, readily passes through the placenta, either via passive diffusion or by binding to a specific carrier [30]. Once it enters the fetal bloodstream, CO binds to hemoglobin, resulting in carboxyhemoglobin. This complex is unable to release oxygen into the fetal tissues, causing systemic hypoxia with consequent delayed maturation in all the organs, especially those most susceptible to hypoxic damage, including the brain [30,31,32]. 

Cigarette smoking can also alter the composition of breast milk, reducing the intake of long-chain polyunsaturated fatty acids, especially omega-3 fatty acids and docosahexaenoic acid (DHA), which are important for the child’s visual and neurological development. The milk of women who smoke is also low in iodine (which is essential for the formation of thyroid hormones in infants), vitamins (especially vitamin C) and antioxidant factors, thus decreasing protection of the infant against infectious agents [33,34,35,36].

## 3. Anthropogenic Chemical Pollution

Anthropogenic chemical pollution refers to contamination of the natural environment by chemicals produced during human activity. These chemicals can be released into the air, water or soil, and can have a variety of negative impacts on human health [37].

One of the most common sources of anthropogenic chemical pollution is the use of pesticides [38]. These pollutants, when released into the environment, can contaminate food, water and soil. Industrial processes are another important source of anthropogenic chemicals, many of which are released into the environment during the manufacturing process. The pharmaceutical industry is particularly responsible for emissions of volatile organic compounds, which easily evaporate into the air; as such, they contribute to the formation of smog and ground-level ozone, both of which can severely harm human health. Damage can also be caused by the use of pharmaceuticals themselves, as they are often excreted in the urine, thus contaminating wastewater. Pharmaceuticals can also be released into the environment through landfill and incinerators. 

In recent decades, researchers have also acknowledged the fundamental toxic role of nanomaterials (i.e., ultrafine particles with dimensions under 100 nm), which are widely used in biomedicine, biotechnology and the environmental industry [39,40]. Therefore, nanotechnology, which are increasingly employed due to their potential in the creation of new therapies and diagnostic tools, could be considered a serious hazard to human health [41].

Some of the most common effects of anthropogenic chemical pollution on human health include respiratory pathologies (such as asthma, bronchitis and pneumonia), cancer, birth defects, neurological problems, immune system alterations and endocrine disruption. Endocrine disruptors, which consist of a vast, heterogeneous group of molecules and/or mixtures of environmental toxic substances that can alter the normal hormonal functionality of the endocrine system, deserve particular attention. 

## 4. Endocrine Disruptor Chemicals (EDCs)

Endocrine disruptor chemicals (EDCs), regardless of their chemical nature, have the ability to interfere with the natural biological function of hormones by enabling, disabling or modifying their signals, thus leading to a wide range of specific pathologies (such as infertility, metabolic alterations, immune deficiencies, thyroid dysfunction, diabetes mellitus and hormone-dependent tumors) [42,43]. 

Pesticides, brominated flame retardants, plasticizers and many other industrial products belong to this group. Examples of endocrine disruptors include:

- Bisphenol A (BPA), which occurs in some food storage containers;
- Dioxin, which is mainly produced during production processes involving the burning of specific substances and in the initial stages of waste combustion;
- Perfluoroalkyl and Polyfluoroalkyl Substances (PFAS), which are used in non-stick coatings;
- Phthalates, which are used to make plastics more pliable;
- Polychlorinated biphenyls (PCBs), which are mainly present in transformers and lubricants;
- Triclosan, which is found in many antibacterial products;
- Pesticides used in agriculture (e.g., organochlorine, organophosphate and carbamate pesticides);
- Polybrominated diphenyl ethers, which are mixtures of chemicals that are added to a wide variety of products to make them less flammable.

Some of these EDCs (e.g., BPA, phthalates or dioxins) are known as obesogens due to their ability to promote obesity [10].

EDCs also include several synthetic chemicals with which we may come into contact every day, as they occur in personal care and/or household cleaning products, vast quantities of which are released on the market every year. Furthermore, it is well known that a minority of these products have been tested for health effects [44]. 

## 5. Gene–Environment Interactions

The study of the interaction of an individual’s genetic constitution with various environmental factors represents one of the most promising current research areas for understanding many multifactorial and chronic pathologies, such as cancer, obesity and neurodegenerative diseases [45,46]. In-depth molecular research on the interaction between DNA and the environment could lead to the identification of predictive therapeutic and prognostic biomarkers, and therefore, individuals most at risk of health problems in the general population.

In conclusion, common chronic diseases, such as cancer, cardiovascular disease, diabetes and obesity, can result from repeated exposure to pollutants over time, in addition to being related to individual genetic constitution. The protection of human health from the effects of environmental exposure is a serious public health concern. Understand how individual pollutants interact with the biological processes of our cells is crucial to the development of effective prevention and intervention strategies. Environmental Exposure Science (EES) is a new scientific discipline that focuses on studying the relationship between the environment and human health [47]. This science encompasses a range of scientific disciplines, including toxicology, epidemiology, environmental chemistry, exposure assessment and risk assessment. EES has two primary goals: (1) to understand how toxic pollutants affect human health and (2) to prevent or reduce contact with these harmful stressors, and thus, improve public health. EES research primarily aims to determine the types, levels and combinations of exposure that people encounter, and the resulting diseases they develop throughout their lives. For the purposes of developing EES research, this Special Issue, entitled “Environmental Exposure Science and Human Health” aims to collect the most innovative research in this field. These contributions should provide impetus to the coordination of global efforts to promote healthy environments.
